# Evaluation of coronary artery disease in congenital heart disease and pediatrics utilizing adenosine stress perfusion

**DOI:** 10.1186/1532-429X-13-S1-P200

**Published:** 2011-02-02

**Authors:** Michael J Campbell, Piers CA Barker, Frederique Bailliard, Ray J Kim

**Affiliations:** 1 Duke University, Durham, NC, USA

## Introduction

The diagnosis of CAD utilizing adenosine stress perfusion is well described in adults. CAD in children is rare but can occur in the setting of congenital and acquired heart disease. Adults with CHD, a growing patient population, are at risk for CAD. A review of the medical literature revealed one previous study involving 4 patients in which adenosine stress perfusion was used for evaluating CAD in these populations.

## Purpose

To evaluate the safety and feasibility of adenosine stress perfusion in the assessment of coronary artery disease (CAD) in congenital heart disease (CHD) and pediatrics.

## Methods

A retrospective chart review was performed on consecutive patients with a diagnosis of CHD or age < 18yo in which adenosine stress perfusion was attempted. SSFP cine and delayed enhancement CMR (DE-CMR) were performed in a standard manner. Adenosine stress perfusion was performed with the administration of 140ug/kg/min of adenosine for 2-4 minutes and 0.1mmol/kg of gadolinium using a standard adult protocol. Patients with abnormal DE-CMR in a pattern consistent with coronary artery distribution were considered to have myocardial infarction (MI). A stress perfusion defect larger than the infarct on DE-CMR was indicative of inducible ischemia.

## Results

32 studies were attempted in 30 patients (mean 31 years, 12 < 18 years). 97% of the studies were completed safely and with images of diagnostic quality. Stress perfusion was discontinued in a patient with Ebstein’s anomaly and atrial flutter who had increased ventricular rates with adenosine. General anesthesia was used in 3 studies. Diagnoses and symptoms are listed in Tables 1 and 2. 35% of studies had evidence of CAD. 26% had DE-CMR evidence of MI. 16% had evidence of inducible ischemia on stress perfusion. 8 patients had a cardiac catheterization, and there was agreement with CMR in 6. A patient with repaired anomalous right coronary artery from the pulmonary artery had abnormal stress perfusion but normal catheterization. A patient with repaired anomalous left coronary artery from the pulmonary artery had a CMR in the post-operative period with evidence of infarct and inducible ischemia but no stenosis on catheterization. A CMR result negative for CAD resulted in no further workup in 18/20 (90%). A finding of CAD on CMR resulted in continued workup or intervention in 9/11 (82%).

**Table 1:** Diagnoses

Tetralogy of Fallot s/p repair	6
Kawasaki disease	6
Anomalous left coronary artery arising from pulmonary artery-repaired	3
Ventricular septal defect s/p repair	2
Coarctation of the aorta s/p surgical repair	1
Coarctation of the aorta s/p transcatheter stent	1
Left coronary artery arising from the right coronary sinus-repaired	1
Right coronary artery arising from the left coronary sinus-repaired	1
Anomalous right coronary artery arising from pulmonary artery-repaired	1
Pulmonary atresia with intact ventricular septum	1
Transposition of the great arteries s/p Mustard operation	1
Right coronary artery aneurysm, etiology unknown	1
Aortic stenosis s/p Ross operation	1
Scimitar syndrome s/p repair and s/p coronary artery bypass graft	1
Congenitally corrected transposition of the great arteries	1
Hypoplastic right pulmonary artery, hypoplastic right coronary artery	1
Dysplastic pulmonary valve with pulmonary insufficiency	1
Sinus venosus atrial septal defect and partial anomalous pulmonary venous return-repaired	1
Ebstein’s anomaly s/p tricuspid valve repair	1

**Table 2:** Presenting Symptom

Chest pain	16
Asymptomatic, screening	7

Abnormal echocardiogram	3

Abnormal electrocardiogram	2

Dyspnea	2

Syncope	1

Nausea and fatigue	1

## Conclusion

CMR with adenosine stress perfusion can be safely performed in CHD and pediatrics. CMR can be used to evaluate CAD and influence outcomes in these populations.

